# Plasmid Mediated *mcr-1.1* Colistin-Resistance in Clinical Extraintestinal *Escherichia coli* Strains Isolated in Poland

**DOI:** 10.3389/fmicb.2021.547020

**Published:** 2021-12-10

**Authors:** Piotr Majewski, Anna Gutowska, David G. E. Smith, Tomasz Hauschild, Paulina Majewska, Tomasz Hryszko, Dominika Gizycka, Boguslaw Kedra, Jan Kochanowicz, Jerzy Glowiński, Justyna Drewnowska, Izabela Swiecicka, Pawel T. Sacha, Piotr Wieczorek, Dominika Iwaniuk, Anetta Sulewska, Radoslaw Charkiewicz, Katarzyna Makarewicz, Agnieszka Zebrowska, Slawomir Czaban, Piotr Radziwon, Jacek Niklinski, Elzbieta A. Tryniszewska

**Affiliations:** ^1^Department of Microbiological Diagnostics and Infectious Immunology, Medical University of Białystok, Białystok, Poland; ^2^Institute of Biological Chemistry, Biophysics and Bioengineering, Heriot-Watt University, Edinburgh, United Kingdom; ^3^Department of Microbiology, Institute of Biology, University of Białystok, Białystok, Poland; ^4^Regional Centre for Transfusion Medicine, Białystok, Poland; ^5^Second Department of Nephrology and Hypertension with Dialysis Unit, Medical University of Białystok, Białystok, Poland; ^6^Second Department of General and Gastroenterological Surgery, Medical University of Białystok, Białystok, Poland; ^7^Department of Neurology, Medical University of Białystok, Białystok, Poland; ^8^Department of Vascular Surgery and Transplantation, Medical University of Białystok, Białystok, Poland; ^9^Department of Clinical Molecular Biology, Medical University of Białystok, Białystok, Poland; ^10^Department of Anesthesiology and Intensive Care, Medical University of Białystok, Białystok, Poland; ^11^Department of Hematology, Medical University of Białystok, Białystok, Poland

**Keywords:** colistin-resistance, IncX4 plasmid, *mcr-1.1*, extraintestinal *E. coli*, plasmid

## Abstract

**Objectives:** The growing incidence of multidrug-resistant (MDR) bacteria is an inexorable and fatal challenge in modern medicine. Colistin is a cationic polypeptide considered a “last-resort” antimicrobial for treating infections caused by MDR Gram-negative bacterial pathogens. Plasmid-borne *mcr* colistin resistance emerged recently, and could potentially lead to essentially untreatable infections, particularly in hospital and veterinary (livestock farming) settings. In this study, we sought to establish the molecular basis of colistin-resistance in six extraintestinal *Escherichia coli* strains.

**Methods:** Molecular investigation of colistin-resistance was performed in six extraintestinal *E. coli* strains isolated from patients hospitalized in Medical University Hospital, Bialystok, Poland. Complete structures of bacterial chromosomes and plasmids were recovered with use of both short- and long-read sequencing technologies and Unicycler hybrid assembly. Moreover, an electrotransformation assay was performed in order to confirm IncX4 plasmid influence on colistin-resistance phenotype in clinical *E. coli* strains.

**Results:** Here we report on the emergence of six *mcr*-1.1-producing extraintestinal *E. coli* isolates with a number of virulence factors. Mobile pEtN transferase-encoding gene, *mcr-*1.1, has been proved to be encoded within a type IV secretion system (T4SS)-containing 33.3 kbp IncX4 plasmid pMUB-MCR, next to the PAP2-like membrane-associated lipid phosphatase gene.

**Conclusion:** IncX4 *mcr-*containing plasmids are reported as increasingly disseminated among *E. coli* isolates, making it an “epidemic” plasmid, responsible for (i) dissemination of colistin-resistance determinants between different *E. coli* clones, and (ii) circulation between environmental, industrial, and clinical settings. Great effort needs to be taken to avoid further dissemination of plasmid-mediated colistin resistance among clinically relevant Gram-negative bacterial pathogens.

## Introduction

The growing incidence of multidrug-resistant (MDR) bacteria is an unavoidable challenge in modern medicine. Constant selection of MDR bacteria significantly contributes to the reduction of available therapeutic options. Colistin, also referred to as polymyxin E, is a cationic polypeptide considered a “last-resort” antimicrobial for treating infections caused by MDR Gram-negative bacterial pathogens, along with carbapenems and tigecycline (Livermore et al., 2011). Colistin was originally introduced in the 1950s for the treatment of infections caused by Gram-negative bacteria; however, polymyxins fell out of favor in the middle of the 1970s due to high rates of nephro- and neurotoxicity coupled with the advent of less toxic antibacterial agents. Nevertheless, by the mid-1990s (El-Sayed Ahmed et al., 2020), polymyxins were reintroduced into clinical practice due to the emergence of extensively drug-resistant (XDR) Gram-negative bacteria, and currently serve a critical role in the antimicrobial armamentarium (Kaye et al., 2016). Moreover, colistin often stands as the last antimicrobial agent retaining activity against carbapenem-resistant *Enterobacteriaceae*, *Pseudomonas aeruginosa*, and *Acinetobacter baumannii* (Olaitan et al., 2014). Unfortunately, bacterial resistance to polymyxin E emerged rapidly, and could potentially lead to essentially untreatable infections, particularly in the hospital setting where aforementioned XDR microorganisms frequently cause life-threatening infections in the most vulnerable patient populations (Kaye et al., 2016).

Colistin is capable of interacting with lipid A moiety of the lipopolysaccharide (LPS), thereby expelling Ca^2+^ and Mg^2+^ ions from phosphate groups and resulting in disruption of the negatively charged outer membrane (OM) of Gram-negative bacteria. Therefore, the ability of bacteria to resist killing by antimicrobial cationic polypeptides often entails modification of the OM (LPS modification resulting in reduced OM net negative charge). Polymyxin resistance has increased gradually within the last few years, and knowledge on a wide variety of possible chromosomal or acquired resistance mechanisms is still expanding (Olaitan et al., 2014). Most mechanisms conferring polymyxin resistance are directed at modifications of the lipid A moiety of the LPS, which is the primary target of colistin. Most genetic alterations, either chromosomal or acquired, entail a common lipid A modification pathway with 4-amino-4-deoxy-L-arabinose (L-Ara4N) and/or phosphoethanolamine (pEtN) addition. The most important target of L-Ara4N is the 4′-phosphate group of lipid A, but it can also be added to the 1-phosphate group or 3-deoxy-D-manno-oct-2-ulosonic acid (Kdo) (Baron et al., 2016). Substitution of the phosphate groups by L-Ara4N is followed by significant reduction of net negative charge of lipid A to 0, while pEtN modifications are associated with a net charge decrease from −1,5 to −1 (Nikaido, 2003). Therefore, L-Ara4N modification seems to be the most effective, owing to the nature of the OM charge modification (Olaitan et al., 2014). In Enterobacteriaceae, the aforementioned modifications of lipid A can result from mutation in the two-component systems (TCSs) such as PhoPQ, BasSR (PmrAB), small feedback-inhibition peptide MgrB, as well as from plasmid-mediated determinants (*i.e.*, *mcr* gene encoding pEtN transferase) (Zeng et al., 2016; Litrup et al., 2017; Roer et al., 2017; Torpdahl et al., 2017; Osei Sekyere, 2019). The first plasmid-mediated polymyxin resistance gene, termed as *mcr*-1 (currently *mcr*-1.1) was identified in China in November 2015, and was subsequently reported all over the world, in both retrospective and prospective studies (Liu et al., 2016). The earliest, so far described, *mcr-*producing strains date back to the end of the previous century, in the 1980s (Shen et al., 2016). The earliest *mcr*-producing bacterial strain of clinical origin was a *Shigella sonnei* isolated from a pediatric patient in Vietnam, in 2008 (Pham Thanh et al., 2016). The various *mcr* variants (*mcr*-1 to *mcr-*10) have been so far, identified in various species of Gram-negative pathogens originating from animals, meat, food products, environmental, and human sources (Partridge et al., 2018; Nang et al., 2019). The emergence of plasmid-mediated pEtN transferase-encoding genes is a matter of serious concern due to the potential for rapid dissemination *via* horizontal gene transfer. Broad distribution of *mcr* genes in multidrug-resistant hospital strains would be especially dangerous in clinical settings, and could possibly result in wide dissemination of pandrug-resistant bacteria and untreatable infections. Here, we report on the emergence of *mcr*-1.1-harboring IncX4 plasmid in six extraintestinal *Escherichia coli* strains of clinical origin isolated in University Hospital of Bialystok, Poland between 2016 and 2018.

## Materials and Methods

### Clinical Isolates Used in the Study

Colistin-resistant *E. coli* isolates were obtained during microbiological screening of infected patients hospitalized in Medical University Hospital in Bialystok, between 2016 and 2018. Extraintestinal solates originated from postoperative wound swab, bedsore swab, perianal abscess swab, pharyngeal swab, bronchial aspirate, and endotracheal tube secretion. Clinical characteristics of the patients colonized by extraintestinal *mcr-*1.1-producing *E. coli* strains are presented in Table 1.

**TABLE 1:** Clinical characteristics of the patients colonized by extraintestinal *mcr-*1.1-producing *E. coli* strains.

Patient 1 M6 ST-553 –:H20	Second Clinic of Nephrology Clinical origin: bedsore swab Date of isolation: 6th September 2017 Diagnosis: end-stage renal disease (lupus nephropathy) Antimicrobial therapy: ceftazidime, ciprofloxacin; Hospitalization: Clinic of General and Gastroenterological Surgery, ICU
Patient 2 M9 ST-6856 O176:H45	**2nd Clinical Department of General and Gastroenterological Surgery** Clinical origin: perianal abscess swab Date of isolation: 5th March 2018 Diagnosis: perianal abscess Antimicrobial therapy: cephazolin, ciprofloxacin, metronizadole; Hospitalization: 1st Clinical Department of General and Endocrine Surgery

Patient 3 M10 ST-162 O126:H45	**Department of Hematology** Clinical origin: pharyngeal swab Date of isolation: 13th March 2018 Diagnosis: philadelphia chromosome-positive chronic myeloid leukemia Antimicrobial therapy: colistin, amikacin, gentamicyn, meropenem, ciprofloxacin, piperacillin with tazobactam, metronidazole, linezolid, vancomycin. Hospitalization: Clinic of Internal and Metabolic Diseases;

Patient 4 M11 ST-10 O89m:H9	**Department of Neurology** Clinical origin: bronchial aspirate Date of isolation: 29th March 2018 Diagnosis: ischemic stroke; hypertension; type-II diabetes; heart failure; Antimicrobial therapy: amoxicillin with clavulanic acid; Hospitalization: none

Patient 5 M12 ST-10 O89m:H10	**Department of Vascular Surgery and Transplantation** Clinical origin: postoperative wound swab Date of isolation: 4th May 2018 Diagnosis: critical ischemia of the left lower limb due to atherosclerosis; Antimicrobial therapy: metronidazole, linezolid. Hospitalization: Vascular Surgery, ICU

Patient 6 M14 ST-93 O7:H4	**2nd Clinical Department of General and Gastroenterological Surgery** Clinical origin: endotracheal tube secretion Date of isolation: 21st July 2016 Diagnosis: entrapment of the femoral hernia; hypertension; ischemic heart disease; Antimicrobial therapy: tetracycline, ciprofloxacin, metronizadole; Hospitalization: none

### Bacterial Identification and Antimicrobial Susceptibility Testing

Bacterial isolates identification was performed with VITEK-MS (bioMérieux, Marcy l’Etoile, France); with subsequent antimicrobial susceptibility testing (AST) using the VITEK 2 system (bioMérieux, Marcy l’Etoile, France), SensiTest Colistin broth microdilution method (Liofilchem, Roseto degli Abruzzi, Italy), and MIC Test Strips (Liofilchem, Roseto degli Abruzzi, Italy) following manufacturer guidelines. AST results were interpreted in accordance with European Committee on Antimicrobial Susceptibility Testing (EUCAST) criteria.

### Whole Bacterial DNA Extraction and Sequencing

Whole bacterial DNA from six clinical extraintestinal *E. coli* was isolated from Luria Broth overnight cultures with use of silica column-based Genomic Mini AX Bacteria kit (A&A Biotechnology). Purified bacterial DNA was sequenced using both short- and long-read methodologies (Illumina and Oxford Nanopore Technology).

In the first step of molecular analysis, Nextera XT library preparation kit and Nextera XT Indexes (Illumina) were used for previously quantified bacterial DNA, which was simultaneously fragmented and tagged with sequencing adapters in a single-tube enzymatic reaction. Quality and quantity of libraries were assessed by fluorometry (Qubit, Thermo Fisher Scientific) and chip electrophoresis (2100 Bioanalyzer, Agilent). FASTQ reads were generated with the use of MiSeq Reagent Kit v3 (600 cycles) and MiSeq analyzer (Illumina).

In the next step, DNA libraries were prepared with the use of a Ligation Sequencing Kit (SQK-LSK109) with Native Barcoding Expansion (EXP-NBD104). Quality and quantity of libraries were assessed by fluorometry (Qubit, Thermo Fisher Scientific) and chip electrophoresis (2100 Bioanalyzer, Agilent). FASTQ reads were generated with the use of Spot-ON Flow Cell (FLO-MIN106D R9 Version) and MinION Mk1b analyzer (Oxford Nanopore Technology).

### Raw Data Quality Assessment and Downstream Bioinformatics

After quality assessment and quality filtering, reads were trimmed (Trimmomatic in case of Illumina reads), and demultiplexed with Porechop in case of long ONT reads (Bolger et al., 2014). Full structures of bacterial chromosomes and plasmids were recovered using Unicycler hybrid assembler which utilizes spades.py, racon, makeblastdb, tblastn, bowtie2, samtools, bcftools, and pilon (Wick et al., 2017). Alignment and mapping of nucleotide sequences were performed using Geneious 10.0.9 software (Biomatters Ltd., Auckland, New Zealand). A RAST (Rapid Annotation using Subsystem Technology)-annotated genomes were subjected to subsequent *in silico* analyses with use of PlasmidFinder, Resfinder, Virulence Finder (Carattoli et al., 2014; Brettin et al., 2015; Bortolaia et al., 2020).

#### Strain Phylogenomics

The final assembled genome sequence data were uploaded to the Type (Strain) Genome Server (TYGS), a free bioinformatics platform available under https://tygs.dsmz.de, for a whole genome-based taxonomic analysis (Meier-Kolthoff and Göker, 2019).

For the phylogenomic inference, all pairwise comparisons among the set of genomes were conducted using the Genome BLAST Distance Phylogeny approach (GBDP) and accurate intergenomic distances inferred under the algorithm ‘trimming’ and distance formula d5 (Meier-Kolthoff et al., 2013). Phylogenomic tree inferred with FastME 2.1.6.1 (Lefort et al., 2015) from GBDP distances calculated from genome sequences.

#### IncX4 Plasmids Phylogenomics

For the purpose of IncX4 plasmid phylogenomic inference, 100 similar sequences from the BLAST database were aligned and analyzed with the use of Clustal Omega (clustalo 1.2.4). Resulting phylogenetic tree was visualized using iTOL v6^1^ (Letunic and Bork, 2021). Structural comparison between colistin-conferring plasmids harbored by studied extraintestinal *E. coli* isolates and IncX4 plasmid sequences deposited in NCBI was prepared using BLAST Ring Image Generator (BRIG) – default parameters with 90/70 as upper/lower threshold (Alikhan et al., 2011).

### Transconjugation Assays

Electrotransformation of the IncX4 plasmid into the recipient *E. coli* strain was performed in order to confirm its influence on colistin-resistance phenotype in clinical *E. coli* isolates. To determine whether *mcr*-1.1 gene was located on pMUB-MCR 33.3 kbp IncX4 plasmid, transconjugation experiments were performed, with plasmid profiles preparation using Plasmid Mini AX kit (A&A Biotechnology), and subsequent electrotransformation into plasmid-free and colistin-sensitive *E. coli* TOP10 strain. Electrotransformation with subsequent selection of the transformants on the Luria-Bertani medium containing 1 mg/L colistin was conducted for the *E*. *coli* TOP10 strain.

## Results

Extraintestinal *E. coli* isolates incorporated into the described study presented a relatively similar antimicrobial resistance pattern (66.66%; 4 of 6). *E. coli* MIN6 ST-553, MIN10 ST-162, MIN11 ST-10, and MIN12 ST-10 were found to be resistant to amoxicillin/clavulanic acid (MIC > 32 mg/L), ciprofloxacin (MIC ≥ 4 mg/L), trimethoprim/sulfamethoxazole (MIC ≥ 320 mg/L), and colistin (MIC = 4 mg/L, except of strain MIN12 – MIC = 8 mg/L). *E. coli* MIN9 ST-6856 was found to be resistant to amoxicillin/clavulanic acid (MIC > 32 mg/L), gentamicin (MIC ≥ 16 mg/L), ciprofloxacin (MIC ≥ 4 mg/L), trimethoprim/sulfamethoxazole (MIC ≥ 320 mg/L), and colistin (MIC = 4 mg/L). Furthermore, *E. coli* MIN14 strain was resistant to amoxicillin/clavulanic acid (MIC > 32 mg/L), and presented the highest colistin MIC = 16 mg/L. Detailed antimicrobial susceptibility testing results of six extraintestinal *E. coli* strains are presented in Table 2.

**TABLE 2:** Antimicrobial susceptibility of extraintestinal *mcr-*1.1-producing *E. coli* strains.

	M6 ST-553 –:H9		M9 ST-6856 O176:H45		M10 ST-162 O126:H45		M11 ST-10 O89:H9		M12 ST-10 O89:H10		M14 ST-93 O7:H4	
Amikacin	≤2	S	≤2	S	≤2	S	≤2	S	≤2	S	≤2	S
Gentamicin	≤1	S	≥16	R	≤1	S	≤1	S	≤1	S	≤1	S
Amoxicillin/Clavulanic acid	≥32	R	≥32	R	16	R	≥32	R	≥32	R	≥32	R
Cefepime	≤0.12	S	≤0.12	S	≤0.12	S	≤0.12	S	≤0.12	S	≤0.12	S
Cefotaxime	≤0.25	S	≤0.25	S	≤0.25	S	−	−	≤0.25	S	≤0.25	S
Cefuroxime	4	S	−	−	4	S	−	−	8	S	4	S
Imipenem	≤0.25	S	≤0.25	S	≤0.25	S	≤0.25	S	≤0.25	S	≤0.25	S
Meropenem	≤0.25	S	≤0.25	S	≤0.25	S	≤0.25	S	≤0.25	S	≤0.25	S
Ciprofloxacin	≥4	R	≥4	R	≥4	R	≥4	R	≥4	R	≤0.25	S
Tigecycline	≤0.5	S	≤0.5	S	−	−	−	−	−	−	≤0.5	S
Trimethoprim/Sulfamethoxazole	≥320	R	≥320	R	≥320	R	≥320	R	≥320	R	≤20	S
Colistin	4	R	4	R	4	R	4	R	8	R	16	R

Whole-genome sequencing with use of a hybrid assembly approach allowed us to recover full genome and mobilome structures of tested extraintestinal colistin-resistant *E. coli* strains. Detailed characteristics of the sequenced genomes are presented in Table 3.

**TABLE 3:** Detailed characteristics of the sequenced *E. coli* genomes.

Strain	Accession no./GenBank sequence	ST	O:H genotype	Coverage (X)	Size (Mb)	No. of contigs	No. of plasmids	%GC	No. of rRNAs	No. of tRNAs	*N*_50_	*L*_50_	No. of coding sequences	No. of CRISPR arrays	Chromosomal MGE	Chromosomal antimicrobial resistance determinants
MIN6	SAMN17831481 CP069692.1	533	−:H20	245	5.187	9	8	50.91	22	86	4,989,845	1	5177	2	44	*mdf(A); gyrA*:p.S83L; *parC*:p.S80I; *gyrA*:p.D87N; *parC*:p.E84G
MIN9	SAMN17831482 CP069682.1	6856	O176:H45	80	5.032	10	9	50.55	22	86	4,590,315	1	5045	2	28	mdf(A); *gyrA*:p.D87N; *gyrA*:p.S83L; *parC*:p.S80I
MIN10	SAMN17831483 CP069677.1	162	O126:H45	260	5.402	5	4	50.56	22	97	5,122,973	1	5389	2	39	*mdf(A);* *gyrA*:p.S83L; *gyrA*:p.D87N;
MIN11	SAMN17831484 CP069666.1	10	O89m:H9	96	4.988	11	10	50.41	22	87	4,768,306	1	4930	2	39	*mdf(A); bla*_*TEM–1A*_; *aadA1; dfrA1;* *parC*:p.S80R; *gyrA*:p.S83L; *gyrA*:p.D87N; *parE*:p.E460D;
MIN12	SAMN17831485 CP069657.1	10	O89m:H10	484	5.274	9	8	50.79	22	87	4,896,568	1	5302	1	48	*mdf(A); tet(B); gyrA*:p.D87N; *parC*:p.S80I; *gyrA*:p.S83L; *parE*:p.S458A
MIN14	SAMN17831486 CP069646.1	93	O7:H4	83	5.032	11	10	50.62	22	90	4,780,475	1	5029	1	28	mdf(A);

Moreover, all six colistin-resistant extraintestinal *E. coli* strains were equipped with a relatively rich plasmidic panel (Table 4). Interestingly, all of the tested strains harbored a IncX4 33.3 kbp plasmid pMUB-MCR, with the mobile pEtN transferase-encoding gene, *mcr-*1.1, which has been proved to be encoded within a type IV secretion system (T4SS), next to the PAP2-like membrane-associated lipid phosphatase gene (Figures 1, 2). The biological consequences of pMUB-MCR IncX4 plasmid possession were evaluated with the use of a transconjugation assay. The *E. coli* TOP10 electrotransformants carrying the 33.3 kbp IncX4 plasmid showed a MIC of colistin of 2 mg/L, which corresponded to a 16-fold increase as compared to the recipient *E. coli* TOP10 strain. These data confirmed that 33.3 kbp IncX4 pMUB-MCR conjugative plasmid is responsible for colistin-resistance in six extraintestinal clinical *E. coli* strains isolated from patients hospitalized in Medical University Hospital in Bialystok, Poland. Moreover, phylogenomics of IncX4 plasmids bearing mobile pEtN transferase-encoding genes is presented in Figure 3.

**TABLE 4:** Plasmids harbored by six extraintestinal colistin-resistant *E. coli* strains.

Strain	Plasmid name	Length (bp)	%GC	Plasmid type	Mobile genetic elements (position in contig)	Content
MIN6	pMUB-MIN6-MCR	33 288	41.84	IncX4	IS26_(20481–21300)_	*mcr*-1.1; *vir*B1; *vir*B3; *vir*B5; *vir*B6; *vir*B8; *vir*B9; *vir*B10; *vir*B11; *cag*12 pathogenicity island; hemolysin expression modulator; *hicAB* toxin/antitoxin;
	pMUB-MIN6-1	89 956	51.50	IncFII	cn_3430_IS26 with *bla*_*TEM–1B(12228*–15658)_; IS26_(14839–15658)_; Tn4352_(10370–13046)_; ISSso4_(60189–62827)_; IS421_(51366–52697)_; cn_9380_IS26_(14838–24218)_	*bla*_*TEM–1B*_; *aph*(3″)-Ib; *aadA1*; *aph*(6)-Id; *tet*(B); *dfrA1*; *sul2*; mercuric resistance; *lutA*; *lutC*;
	pMUB-MIN6-2	56 897	48.08	IncX1	−	*bla*_*TEM–1B*_; mercuric resistance; *aadA1*; *dfrA15*; *sul1*; hemolysin expression modulator;
	pMUB-MIN6-3	6293	43.83	−	−	DNA adenine methylase; RNAI modulator protein Rom;
	pMUB-MIN6-4	5631	47.38	−	−	mobilization protein MobC, mobilization protein MbeD; RNAI modulator protein Rom; mRNA interferase RelE; RelE/StbE;
	pMUB-MIN6-5	2080	43.37	−	−	chaperone protein DnaJ;
	pMUB-MIN6-6	1551	51.52	Col(MG828)	−	*repA* – replication protein-encoding gene
	pMUB-MIN6-7	1506	50.02	Col(MG828)	−	*repA* – replication protein-encoding gene

MIN9	pMUB-MIN9-MCR	33 303	41.85	IncX4	IS26_(860–1679)_	*mcr*-1.1; *vir*B1; *vir*B3; *vir*B5; *vir*B6; *vir*B8; *vir*B9; *vir*B10; *vir*B11; *cag*12 pathogenicity island; hemolysin expression modulator; *hicAB* toxin/antitoxin;
	pMUB-MIN9-1	283 245	47.11	IncHI2A	Tn6024_(97840–130262)_; ISKpn12_(172884–173725)_	*bla*_*TEM–1A*_; *sul1*; *sul2*; *sul3*; *tet*(A); *dfrA1*; *aadA1*; *aac*(3″)-IIa; *aadA2b*; *aph*(3″)-Ib; *aph*(6)- Id; *catA1*; *cmlA1*;
	pMUB-MIN9-2	102 703	47.67	p0111	IS26_(98806–99625)_; IS421_(17495–18833)_; IS30_(32630–33851)_; IS903_(37021–38077)_;	cobalt-zinc-cadmium resistance; AidA-I adhesin-like protein; tet(A);
	pMUB-MIN9-3	5792	46.65	Col440l	−	mobilization protein MobC, mobilization protein mbeD; RNAI modulator protein Rom; mRNA interferase RelE; RelE/StbE;
	pMUB-MIN9-4	5309	51.12	−	−	*aph*(3′)-I; RNAI modulator protein Rom; TnpA transposase; mobilization protein MobC
	pMUB-MIN9-5	4018	53.33	−	−	mobilization protein MobC
	pMUB-MIN9-6	3371	55.15	−	−	mobilization protein MobC; RNAI modulator protein Rom; mobilization protein MbeD
	pMUB-MIN9-7	3191	47.82	−	−	*psp* operon transcriptional activator; *qnrB19*;
	pMUB-MIN9-8	1552	51.87	Col(MG828)	−	*repA* – replication protein-encoding gene

MIN10	pMUB-MIN10-MCR	33 305	41.84	IncX4	IS26_(20365–21184)_	*mcr*-1.1; *vir*B1; *vir*B3; *vir*B5; *vir*B6; *vir*B8; *vir*B9; *vir*B10; *vir*B11; *cag*12 pathogenicity island; hemolysin expression modulator; *hicAB* toxin/antitoxin;
	pMUB-MIN10-1	146 908	50.44	IncFIC(FII)	cn_31050_ISVsa5 with *bla*_*TEM–1B(46166*–77216)_; IS26_(60077–60896)_; ISEc17_(22077–23334)_; IS26_(76249–77068)_; ISVsa5_(115219–116547)_; ISVsa5_(46167–47495)_; IS629_(7713–9008)_; cn_16992_IS26_(60076–77068)_; cn_16992_IS26_(60076–77068)_; cn_23129_ISVsa5_(24366–47495)_;	*bla*_*TEM–1B*_; *adA1*; *sul3*; *dfrA1*; tet(A); *macA*; *macB*; siderophore *iroN*; mercuric resistance operon; aerobactin
	pMUB-MIN10-2	98 084	47.86	p0111	−	*pgmP*; *recT*; Phd-Doc toxin/antitoxin; *parAB*; *pmgL*
	pMUB-MIN10-3	1552	51.87	Col(MG828)	−	*repA* – replication protein-encoding gene

MIN11	pMUB-MIN11-MCR	33 303	41.85	IncX4	IS26_8688–9507_	*mcr*-1.1; *vir*B1; *vir*B3; *vir*B5; *vir*B6; *vir*B8; *vir*B9; *vir*B10; *vir*B11; *cag*12 pathogenicity island; hemolysin expression modulator; *hicAB* toxin/antitoxin;
	pMUB-MIN11-1	85 440	50.2	IncFII	IS26_(53084–53903)_; ISEc31_(83647–84904)_; IS26_(30158–30977)_; IS629_(7713–9022)_; ISEc32_(70540–71719)_; cn_1081_IS26_(52822–53903)_; cn_2984_IS26_(53083–56067)_; cn_7228_IS26_(30157–37385)_;	VapB-VapC toxin/antitoxin; PemL-PemK toxin/antitoxin; *tetR; tet(A)*; permease of the drug/metabolite transporter (DMT) superfamily; RepFIB replication protein A; transcription activator mig-14; outer membrane protease OmpT; InsO; stability (stb) locus of IncFII plasmid NR1; colicin-M; microcin-M; resolvase; *yuaX*; arsenical resistance operon repressor; integron integrase *IntI1*; *ant*(3″)-Ia; phosphoserine phosphatase; *ant*(3″)-Ia; *cmlA1* (MFS efflux pump); *ant*(3′)-I; mercuric resistance operon regulatory protein MerR; *sitABCD* (hydrogen peroxide resistance)
	pMUB-MIN11-2	74 912	49.7	IncFII(pCoo)	−	Phd-Doc toxin/antitoxin; plasmid SOS inhibition proteins PsiA and PsiB; *repA2*; *tetR; tet(B); traM; traY; traJ; traA; traB; traP; traU; traQ; traG; traS; traX; traK; traV; traR; trbA; trbE; trbI; trbC; trbB; trbJ; traW; traF; traH; traT; traD; yihA; finO; traL traC; traI;*
	pMUB-MIN11-3	5874	47.53	−	−	mobilization protein MobC; RNAI modulator protein Rom; Permease of the drug/metabolite transporter (DMT) superfamily
	pMUB-MIN11-4	5514	45.96	−	−	mRNA interferase RelE;RelB/StbD replicon stabilization protein (antitoxin to RelE/StbE)
	pMUB-MIN11-5	5433	47.01	−	−	mobilization protein MobC; RNAI modulator protein Rom
	pMUB-MIN11-6	4286	42.23	Col440I	−	DNA-cytosine methyltransferase
	pMUB-MIN11-7	2089	47.2	Col(BS512)	−	replication protein;
	pMUB-MIN11-8	1552	51.87	Col(MG828)	−	*repA* – replication protein-encoding gene
	pMUB-MIN11-9	1506	50.27	Col(MG828)	−	*repA* – replication protein-encoding gene

MIN12	pMUB-MIN12-MCR	33 303	41.85	IncX4	IS26_(8688–9507)_	*mcr*-1.1; *vir*B1; *vir*B3; *vir*B5; *vir*B6; *vir*B8; *vir*B9; *vir*B10; *vir*B11; *cag*12 pathogenicity island; hemolysin expression modulator; *hicAB* toxin/antitoxin;
	pMUB-MIN12-1	163 427	50.62	IncFII	Tn4352_(55106–57785)_; Tn4352_(72038–74717)_; Tn4352_(69216–71895)_; Tn4352_(66394–69073)_; Tn4352_(63572–66251)_; Tn4352_(60750–63429)_; Tn4352_(57928–60607)_; IS26_(74860–75679)_; IS26_(84711–85530)_; IS26_(30158–30977)_; IS26_(77059–77878)_; ISEc31_(161634–162891)_; IS629_(7713–9022)_; ISEc32_(144647–145826)_; IS102_(127162–128218)_; cn_2957_IS26(30157–33114); cn_1782_IS26_(73897–75679)_; cn_2199_IS26_(74859–77058)_; cn_1159_IS26_(76719–77878)_; cn_8472_IS26_(77058–85530)_;	*bla*_*TEM–1B*_; *repFIB*; ompT; colicin M; microcin M; *vapB*; arsenic resistance operon; *dfrA; aph*(6)-Id; *aph*(3″)-Ia; *cmlA; aph*(3)-I; *tetA; tetR; pemKI; traA; traB; traC; traD; traE; traF; traG; traH; traI; traJ; traK; traL; traP; traR; traS; traT; traQ; traU; traV; traW; traX; traY; trbA; trbB; trbC; trbD; trbE; trbG; trbI; trbJ; trbN;*
	pMUB-MIN12-2	95 526	53.35	IncB/O/K/Z	Tn2_(36309–41258)_; ISVsa3_(31313–32289)_;	*bla*_*TEM–1B*_; Phd-Doc toxin/antitoxin; *psiAB; virD2; floR; aph*(6)-Id; *aph*(3)-I; *pilM; pilV; pilS; pilQ; traB; traU; traW; traS;*
	pMUB-MIN12-3	61 257	51.49	IncFII(pCoo)	−	Phd-Doc toxin/antitoxin; plasmid SOS inhibition proteins PsiA and PsiB; *repA2; finO; tet(B); tetR; traM; traY; traA; traL; traE; trbD; traR; traC; traW; trbC; traN; trbE; trbA; trbB; traH; traD; traK; traP; trbG; traV; trbI; traU; trbC; traQ; trbJ; traH; traS; traT; traB; traU; traN; traF; trbB; traH; traG; traI; traX;*
	pMUB-MIN12-4	12 696	60.37	−	−	resolvase;
	pMUB-MIN12-5	5874	47.53	−	−	mobilization protein MobC; RNAI modulator protein Rom; permease of the drug/metabolite transporter (DMT) superfamily; mobilization protein MbeD
	pMUB-MIN12-6	3897	51.78	Col156	−	*repA* – replication protein-encoding gene; mobilization protein;
	pMUB-MIN12-7	2255	42.75	Col(MG828)	−	ORF8;

MIN14	pMUB-MIN14-MCR	33 290	41.85	IncX4	IS26_(6158–6975)_	*mcr*-1.1; *vir*B1; *vir*B3; *vir*B5; *vir*B6; *vir*B8; *vir*B9; *vir*B10; *vir*B11; *cag*12 pathogenicity island; hemolysin expression modulator; *hicAB* toxin/antitoxin;
	pMUB-MIN14-1	89 674	48.07	−	−	Phd-Doc toxin/antitoxin; RelE/StbE toxin/antitoxin; phage DNA binding protein Roi; phage baseplate hub; *gp7; gp6*; DNA recombination-dependent growth factor RdgC; Chromosome partitioning protein ParA; ATP-dependent Clp protease ATP-binding subunit ClpX; *pmgC; pmgB; lydB*; phage tail fiber protein (long tail fiber); phage serine/threonine protein phosphatase NinI; heat shock protein C; replication initiation protein RepE;
	pMUB-MIN14-2	67 974	51.42	IncFII(pCoo)	Tn801 with *bla*_*TEM–1D(2133*–7081)_;	*bla*_*TEM–1D*_; Phd-Doc toxin/antitoxin; plasmid SOS inhibition proteins PsiA and PsiB; *repA2*; stable plasmid inheritance protein B; *tet(B); tetR*; X polypeptide; *traM; traJ; traY; traA; traL; traE; traK; traB; traP; trdB; trbG; traV; traR; traC; trbI; traW; traU; trbC; traN; trbE; traF; trbA; traQ; trbB; trbJ; traH; traG; traS; traT; traD; traI; traX; finO;*
	pMUB-MIN14-3	45 893	50.70	IncN	Tn2 with *bla*_*TEM–1B(9973*–14916)_; ISKpn19_(4298–7148)_;	*bla*_*TEM–1B*_; replication initiation protein RepE; RND efflux system, inner membrane transporter; phage DNA invertase; resolvase/integrase Bin; phage integrase, site-specific serine recombinase; IncN plasmid KikA protein; T4SS – *virB; virB3; virB5; virB8; virB10; virB11*; antirestriction protein ArdA; error-prone repair protein UmuD; error-prone, lesion bypass DNA polymerase V (UmuC)
	pMUB-MIN14-4	4091	49.57	Col8282	−	plasmid replication initiation protein
	pMUB-MIN14-5	3688	51.41	−	−	mobilization protein MobC; RNAI modulator protein Rom; mobilization protein MbeD
	pMUB-MIN14-6	2553	44.42	Col440I	−	9.4 kDa protein
	pMUB-MIN14-7	1565	51.05	Col(MG828)	−	*repA* – replication protein-encoding gene
	pMUB-MIN14-8	1552	51.87	Col(MG828)	−	*repA* – replication protein-encoding gene
	pMUB-MIN14-9	1507	50.23	Col(MG828)	−	*repA* – replication protein-encoding gene

**FIGURE 1:**
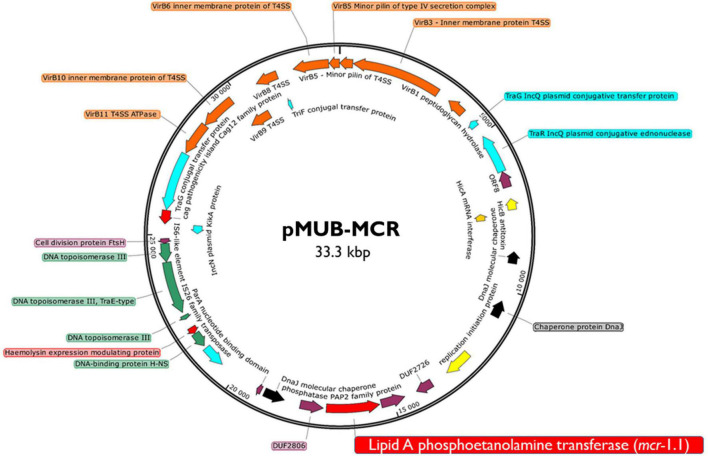
Genetic structure of *mcr*-1.1 harboring IncX4 plasmid. Figure represents a RAST (Rapid Annotation using Subsystem Technology)-annotated plasmid produced by the *E. coli* MIN6 strain. Arrows on the diagram represents annotated functional genes present within the structure of 33.3 kb IncX4 plasmid.

**FIGURE 2:**
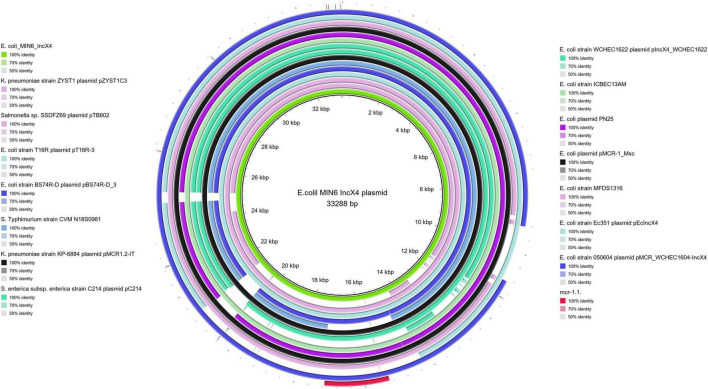
BRIG alignment of IncX4 plasmids recovered from different Enterobacterales. Structural comparison between colistin-conferring plasmids harbored by studied extraintestinal *E. coli* isolates and IncX4 plasmid sequences deposited in NCBI was prepared using BLAST Ring Image Generator (BRIG). The alignment includes the *mcr*-1.1. sequence (red fragment), and the one IncX4 mcr-1–bearing plasmid harbored by *E. coli* MIN6 described in our study (green inner circle).

**FIGURE 3:**
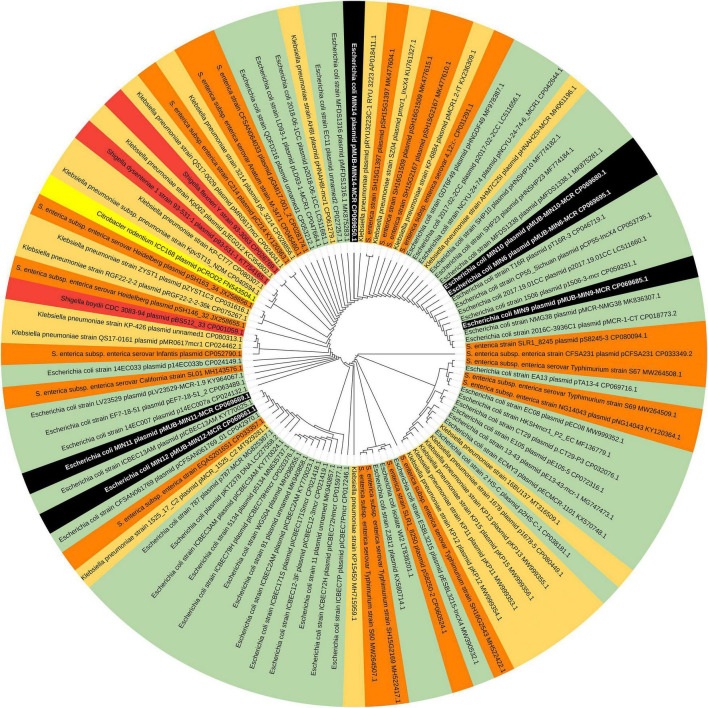
Phylogenomics of IncX4 plasmids bearing mobile pEtN transferase-encoding genes. Colored segments represents bacterial genera harboring IncX4 plasmids – black segments – *E. coli* strains MIN6, MIN9, MIN10, MIN11,MIN12, MIN14; green segments – *E. coli;* light orange segments – *K. pneumoniae;* orange segments – *Salmonella* spp.; red segments – *Shigella* spp., yellow segments – *Citrobacter* spp.

In addition to the IncX4 33.3 kbp plasmid pMUB-MCR, the tested *E. coli* strains were equipped with relatively rich plasmid panels. *E. coli* MIN6 possessed seven plasmids, two of which constituted a vehicle for antimicrobial resistance determinants, namely pMUB-MIN6-1 (IncFII plasmid), and pMUB-MIN-6-2 (IncX1 plasmid). *E. coli* MIN9 harbored eight plasmids, four of which constituted a vehicle for antimicrobial resistance determinants, namely pMUB-MIN9-1 (IncHI2A), pMUB-MIN9-2 (p0111), pMUB-MIN9-4, and pMUB-MIN9-7. *E. coli* MIN10 possessed three plasmids, one of which constituted a vehicle for antimicrobial resistance determinants, namely pMUB-MIN10-1 – IncFIC(FII). Among nine plasmids harbored by *E. coli* MIN11, two were responsible for antimicrobial resistance determinants carriage, namely pMUB-MIN-11-1 (IncFII), and pMUB-MIN-11-2 [IncFII(pCoo)]. *E. coli* MIN12 harbored seven plasmids, three of which possessed antimicrobial resistance determinants, namely pMUB-MIN12-1 (IncFII), pMUB-MIN12-2 (IncB/O/K/Z), and pMUB-MIN12-3 [IncFII(pCoo)]. Furthermore, among nine plasmids possessed by *E. coli* MIN14, two harbored antimicrobial resistance genes, namely pMUB-MIN14-2 [IncFII(pCoo)], and pMUB-MIN14-3 (IncN). Detailed features of plasmids harbored by studied *E. coli* strains are presented in Table 4.

**FIGURE 4:**
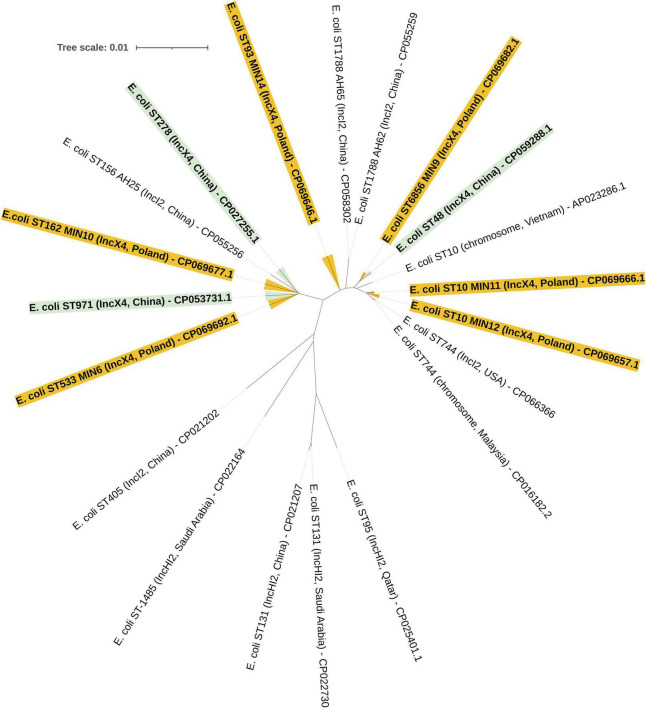
Whole-genome sequence-based GBDP tree of *mcr*-producing *E. coli* complete genomes available at NCBI. Tree inferred with FastME 2.1.6.1 [6 (Lefort et al., 2015)] from GBDP distances calculated from genome sequences. The branch lengths are scaled in terms of GBDP distance formula d5.

The MLST approach was utilized in order to evaluate the molecular relatedness of tested extraintestinal colistin-resistant *E. coli* strains. Among 6 tested clinical strains, two were proven to be clonally related, namely *E. coli* MIN11 and MIN12, which belonged to ST-10. Those two strains were isolated within an interval of 36 days, in the Department of Neurology (MIN11) and Department of Vascular Surgery and Transplantation (MIN12). However, those two strains were easily distinguished by O:H genotype (MIN11 – O89m:H9 vs., MIN12 – O89m:H10), plasmid content (MIN11 – 10 plasmids vs. MIN12 – 8 plasmids), number of chromosomal mobile genetic elements (MIN11 – 39 vs. MIN12 – 48), virulence factors (MIN-12 was distinguished by the presence of heat-stable toxin EAST-1) and content of chromosomal resistance determinants. The remaining extraintestinal colistin-resistant *E. coli* strains belonged to ST-533 (*E. coli* MIN6), ST-6856 (*E. coli* MIN9), ST-162 (*E. coli* MIN10), and ST-93 (*E. coli* MIN14). Moreover, whole-genome sequence-based phylogenomics of colistin-resistant *E. coli* strains is presented in Figure 4.

### Beta-Lactam Resistance

In the present study, all tested strains presented isolated resistance to amoxicillin-clavulanate. Three strains (MIN6, MIN12, MIN14) were equipped with multiple *bla*_*TEM–1*_ genes carried by various plasmids. *E. coli* MIN6 and MIN12 strain harbored duplicate *bla*_*TEM–1B*_ genes, within two distinct mobile vectors, namely pMUB-MIN6-1 (89 956 bp), and MUB-MIN6-2 (56 897 bp) in *E. coli* MIN6, and pMUB-MIN12-1 (163 427 bp), and pMUB-MIN12-2 (95 526 bp) in *E. coli* MIN12. *E. coli* MIN14 possessed two TEM variants, namely *bla*_*TEM–1D*_ and *bla*_*TEM–1B*_ harbored by pMUB-MIN-14-2 (67 974 bp), and pMUB-MIN-14-3 (45 893 bp), respectively. Moreover, E. *coli* MIN9 possessed a single *bla*_*TEM–1a*_ gene within the pMUB-MIN9-1 plasmid (283 245 bp). Interestingly, in case of *E. coli* MIN11, *bla*_*TEM–1B*_ gene was present within the bacterial chromosome structure.

### Fluoroquinolone Resistance

All tested *E. coli* strains, except MIN14, were ciprofloxacin-resistant due to multiple mutations of *gyrA* (p.S83L – 5/5 strains; p.D87N – 5/5 strains), *parC* (p.S80I – 3/5 strains; p.E84G – 1/5 strain; p.S80R – 1/5 strain), and *parE* (p.E460D – 1/5 strain; p.S458A – 1/5 strain) genes, coupled with additional acquired fluoroquinolone resistance gene, *qnrB19*, in case of MIN9 strain (pMUB-MIN9-7). The only ciprofloxacin-susceptible strain MIN14, possessed a plasmid-borne *qnrS1* gene, which could be associated with low ciprofloxacin MICs.

### Aminoglycoside Resistance

All tested *E. coli* strains were amikacin-susceptible, while *E. coli* MIN9 was the only tested strain that presented phenotypic resistance against gentamicin (MIC ≥ 16 mg/L), due to presence of *aac*(3″)-IIa (gene conferring resistance to gentamicin, apramycin, tobramycin, dibekacin, netilmicin, sisomicin) within pMUB-MIN9-1 (InCHI2A). Furthermore, all tested strains except MIN14, produced various aminoglycoside-resistance factors conferring resistance to spectinomycin, streptomycin *[aadA1*; *aadA2b*; *aph*(6)-Id; *aph*(3″)-Ib]; neomycin, kanamycin, lividomycin, paromomycin, ribostamycin (*aph*(3′)-Ia).

### Folate Pathway Antagonist Resistance

All tested *E. coli* strains, except MIN14, presented trimethoprim/sulfamethoxazole-resistance, in accordance with WGS data screening for antimicrobial resistance determinants. *E. coli* MIN11 possesses the chromosomal trimethoprim-resistance gene, *dfrA1*, coupled with plasmidic sulfamethoxazole-resistance gene *sul3.* Moreover, remaining strains harbor plasmidic resistance genes, such as, *dfrA1, dfrA14*, *dfrA15, sul1, sul2*, and *sul3.*

### Phenicol Resistance

*Escherichia coli* MIN9, MIN11, and MIN12 were also equipped with acquired genes conferring resistance to phenicols, namely chloramphenicol acetyltransferase gene *catA1* (pMUB-MIN9-1), and MFS transporters *cmlA1* (pMUB-MIN9-1; pMUB-MIN11-1; pMUB-MIN12-1) and *floR* (pMUB-MIN12-2).

Extraintestinal *E. coli* strains also possess a number of virulence factors, such as long polar fimbriae, heat-stable toxin EAST-1 or enterobactin siderophore. All genes encoding virulence factors are listed in Table 5.

**TABLE 5:** Virulence factors in six extraintestinal colistin-resistant *E. coli* strains.

Strain	Source	Hospital ward	Virulence factors	
			Gene	Function
M6, ST553, –:H20	bedsore swab	Second Clinic of Nephrology	*gad* – glutamate decarboxylase	survival for at least 2 h in a strongly acidic environment
			*iss* – increased serum survival	increased complement resistance
			*lpfA* – long polar fimbriae	adhesive factor contributing to intestine colonization
M9, ST6856, O176:H11	abscess swab	Second General Surgery Clinic	gad – glutamate decarboxylase	survival for at least 2 h in a strongly acidic environment
M10, ST162, O126:H45	pharyngeal swab	Hematology Clinic	astA – heat-stable toxin EAST-1	activation of membrane-bound guanylate cyclase, intracellular accumulation of cGMP
			gad – glutamate decarboxylase	survival for at least 2 h in a strongly acidic environment
			iroN – enterobactin siderophore	acquiring iron for microbial systems
			iss – increased serum survival	increased complement resistance
			lpfA – long polar fimbriae	adhesive factor contributing to intestine colonization
			mchF – ABC transporter protein	antibiotic peptide (microcin) exporter
M11, ST10, O89m:H9	bronchial aspirate	Neurology Clinic	cma – colicin M	inhibition of peptidoglycan and O-antigen biosynthesis
			gad – glutamate decarboxylase	survival for at least 2 h in a strongly acidic environment
M12, ST10, O89m:H10	wound swab	Vascular Surgery Clinic	astA – heat-stable toxin EAST-1	cGMP accumulation and loss of electrolytes and water from intestinal cells
			cma – colicin M	inhibition of peptidoglycan and O-antigen biosynthesis
			gad – glutamate decarboxylase	survival for at least 2 h in a strongly acidic environment
M14, ST93, O7:H4	endotracheal tube secretion	Second General Surgery Clinic	astA – heat-stable toxin EAST-1	cGMP accumulation and loss of electrolytes and water from intestinal cells
			gad – glutamate decarboxylase	survival for at least 2 h in a strongly acidic environment
			iss – increased serum survival	increased complement resistance

## Discussion

In this paper we sought to investigate the mechanism of colistin-resistance in six extraintestinal *E. coli* strains isolated from patients hospitalized in Medical University Hospital, Bialystok, Poland. Full structures of bacterial chromosomes and plasmids were recovered with use of both short- and long-read sequencing technologies and Unicycler hybrid assembly. Results of antimicrobial resistance testing were in accordance with genomic and mobilome screening for antimicrobial resistance determinants. All tested extraintestinal *E. coli* strains harbored an IncX4 33.3 kbp plasmid pMUB-MCR, with the mobile pEtN transferase-encoding gene, *mcr-*1.1. Moreover, its influence on colistin-resistance phenotype was confirmed by transconjugation assays.

In the present study, we report the first detailed description of *mcr*-containing IncX4 plasmid harbored by clinical *E. coli* strains in Poland. In six extraintestinal *E. coli* strains, *mcr-*1.1 was found within a type IV secretion system (T4SS) contained within a 33.3 kbp IncX4 plasmid that is known to be involved in the disseminating of multiple *mcr* variants. It is widely accepted that some type IV secretion systems (T4SSs) in pathogenic Gram-negative bacteria are utilized in order to translocate virulence factors into the host cell, mediate downregulation of the hosts innate immune response genes and an increase bacterial uptake and survival within macrophages and epithelial cells (Gokulan et al., 2013). Moreover, T4SSs could be also responsible for horizontal gene transfer (Juhas et al., 2008), thus contributing to genome plasticity and the evolution of pathogens through dissemination of antibiotic resistance and virulence genes (Juhas et al., 2008). Conjugative T4SSs are often encoded on self-transmissible plasmids coupled with genes that provide selective advantages for the cell such as antibiotic resistance, virulence factors or other metabolic functions that enhance survival (Wallden et al., 2010). The 33 kbp IncX4 plasmid was proven to be highly transmissible, showing 10^2^–10^5^-fold higher transfer frequencies relative to epidemic IncFII plasmid (Lo et al., 2014; Xavier et al., 2016). Moreover, Lo and colleagues proved that 33 kbp IncX4 plasmid carriage is associated with relatively low fitness cost, which makes it a highly effective vehicle for drug resistance determinants (Wu et al., 2018). Interestingly, it has been also recently reported that IncX4 plasmid can be relatively easily and stably maintained in host bacteria (Beyrouthy et al., 2017). In fact, IncX4 plasmids have been recently shown to harbor multiple *mcr* variants, CTX-M extended spectrum β-lactamase, as well as the 33.3 kbp IncX4 vehicles without any drug-resistance determinants (Lo et al., 2014; Chen et al., 2019). Similar IncX4 *mcr-*containing plasmids are reported as increasingly disseminated mainly among *E. coli* isolates (Table 6), suggesting that it is becoming an “epidemic” plasmid, responsible for (i) disseminating colistin-resistance determinants between different *E. coli* clones, and (ii) circulating between environmental, industrial, and clinical settings. The phylogenomics of IncX4 plasmids bearing mobile pEtN transferase-encoding genes is presented in Figure 3.

**TABLE 6:** Global dissemination of *mcr*-harboring 33.3 kbp IncX4 plasmid.

	Origin	Organism	Country	References
*mcr-*1.9	ABF	*E. coli* (swine)	Portugal	Manageiro et al., 2019
*mcr-*1.1	ABF	*E. coli* ST-48; ST-131; ST-359; ST-1112; ST-2063 (chicken)	Denmark	Hasman et al., 2015
*mcr-*1.1	clinical	***S* . Typhimurium ST-34**	United Kingdom	Doumith et al., 2016
*mcr-*1.1	ABF, clinical	***Salmonella* spp.**	Portugal	Campos et al., 2016
*mcr-*1.2	clinical	***K. pneumoniae* ST-512**	Italy	Di Pilato et al., 2016
*mcr-*2	ABF	*E. coli* ST-10 (swine)	Belgium	Xavier et al., 2016
*mcr-*1.2	natural environment	*E. coli* ST-10 (river)	Italy	Caltagirone et al., 2017
*mcr-*1.1 *mcr-*2	swine and poultry meat	*Salmonella* spp.	Belgium	Garcia-Graells et al., 2018
*mcr-*1.2	clinical	***E. coli* ST-354**	Italy	Simoni et al., 2018
*mcr-*1.1	ABF	*E. coli* ST-34; ST-757; ST-1494 (pig slurry)	Estonia	Brauer et al., 2016
*mcr-*1.1	ABF	*E. coli* ST-10 (boot swab); ST-1140 (boot swab); ST-1011 (stable fly); ST-342 (manure); ST-10 (barn dog feces);	Germany	Guenther et al., 2017
*mcr-*1.1	clinical	***E. coli* ST-1288**	France/Portugal	Beyrouthy et al., 2017
*mcr-*1.1	ABF	*E. coli* ST-641 (swine feces)	Germany	Pulss et al., 2017
*mcr-*1.1	clinical	***E. coli* ST-744**/O89:H10	Portugal	Tacão et al., 2017
*mcr-*1.1 *mcr-*1.2	chicken retail meat, clinical	***E. coli* ST-5***;* ST-58	Switzerland	Donà et al., 2017
*mcr-*1.1	ABF, clinical, turkey and chicken meat	***E. coli* ST-48;** ST-58; ST-156; ST-1431 (turkey);	Switzerland	Zurfluh et al., 2017
*mcr-*1.1	ABF	*E. coli* ST-58; ST-69; ST-354; ST-453; ST-1081; ST-1196; ST-5956 (turkey)	Czech Republic	Gelbíčová et al., 2019
		*E. coli* ST-10; ST-93; ST-410; ST-744; ST-746; ST-1385 (turkey)	Poland	
		*E. coli* ST-58; ST-162; ST1011 (turkey)	Germany	
*mcr-*1.1	clinical	***K. pneumoniae* ST-45; ST-1112**	Portugal	Mendes et al., 2018
*mcr-*1.1	clinical	***E. coli* ST-93**	Finland	Gröndahl-Yli-Hannuksela et al., 2018
*mcr-*1.1	ABF	*E. coli* ST-10; ST-48; ST-58; ST-69; ST-88; ST-90; ST-93; ST-117; ST-155; ST-156; ST-162; ST-191; ST-349; ST-354; ST-359; ST-533; ST-602; ST-617; ST-624; ST-919; ST-949; ST-1167; ST-1170; ST-1196; ST-1564; ST-1611; ST-1851; ST-2001; ST-2509; ST-2556; ST-6286; ST-7315 (turkey); *E. coli* ST-37; ST-48; ST-57; ST-86; ST-189; ST-398; ST-1011; *E. coli* ST-1303 (broiler); *E. coli* ST-359 (laying hen); *E. coli* ST-767 (pig)	Poland	Zaja̧c et al., 2019
*mcr-*1.1	ABF	*Salmonella* infantis (broilers);	Italy	Carfora et al., 2018
*mcr-*1.1	ABF	*E. coli* ST-1; ST-10; 118; 4274 (swine)	Spain	García-Meniño et al., 2019
*mcr-*1.1	clinical	***E. coli* ST-2448; *K. pneumoniae* ST-25**	China	Li et al., 2016
*mcr-*1.1	clinical	***E. coli* ST-101**	Brazil	Fernandes et al., 2016
*mcr-*1.1	ABF	*E. coli* ST-1114; *E. coli* ST-167; *E. coli* ST-410; *E. coli* ST-90; *E. coli* ST-4429; *E. coli* ST-4656; *E. coli* ST-156; *E. coli* ST-54; *E. coli* ST-4463; *E. coli* ST-3331; *E. coli* ST-165; *E. coli* ST-1178; *E. coli* ST-1437; *E. coli* ST-2439; *E. coli* ST-48	China	Kong et al., 2017
*mcr-*1.1	wild birds	*E. coli* ST-10	Brazil	Sellera et al., 2017
*mcr-*1.1	clinical	***E. coli* ST-167; *E. coli* ST-10; *E. coli* ST-2973; *E. coli* ST-354; *E. coli* ST-3028; *E. coli* ST-354; *E. coli* ST-156; *E. coli* ST-1011; *E. coli* ST-393; *E. coli* ST-117;*E. coli* ST-69; *E. coli* ST-218; *E. coli* ST-1193; *E. coli* ST-853; *E. coli* ST-58; *E. coli* ST-44;*E. coli* ST-131; *E. coli* ST-117; *E. coli* ST-457**	China	Quan et al., 2017
*mcr-*1.1	clinical	***Salmonella* Typhimurium**	China	Cui et al., 2017
*mcr-*1.1	ABF	*E. coli* ST-48; *E. coli* ST-4419;	Brazil	Monte et al., 2017b
*mcr-*1.1	clinical	*E. coli* O157:H48	United States	Lindsey et al., 2017
*mcr-*1.1	natural environment	*E. coli* ST-1638; *E. coli* ST-46; *E. coli* ST-10; *E. coli* ST-101	Brazil	Fernandes et al., 2017
*mcr-*1.1	ABF	*E. coli* ST-74; *E. coli* ST-1850 (commercial chicken meat)	Brazil	Monte et al., 2017a
*mcr-*1.1	vegetables	*E. coli* ST-48 (lettuce)	China	Luo J. et al., 2017
*mcr-*1.1	hospital environment	*E. coli* ST-10; *E. coli* ST-410 (hospital sewage water)	China	Zhong et al., 2018
*mcr-*1.1	ABF	*E. coli* ST-155; *E. coli* ST-117 (chicken meat imported from Brazil)	Japan	Nishino et al., 2017
		*E. coli* ST-10 (pork meat imported from Spain)		
*mcr-*1.1	hospital environment	*E. coli* ST-1196; *E. coli* ST-165; *E. coli* ST-10; *E. coli* ST-155	China	Zhao et al., 2017
*mcr-*1.1	clinical	***K. pneumoniae* ST-437**	Brazil	Dalmolin et al., 2018
*mcr-*1.1	clinical	***E. coli* ST-10; *E. coli* ST-46; *E. coli* ST-167; *E. coli* ST-410; *E. coli* ST-3944;**	China	Luo Q. et al., 2017
*mcr-*1.1	clinical	***E. coli* ST-201; *E. coli* ST-486**	China	Chan et al., 2018
*mcr-*1.1	clinical	***K. pneumoniae* ST-16; *K. pneumoniae* ST-45**	Thailand	Srijan et al., 2018
*mcr-*1.1	ABF	*E. coli* ST-443	Brazil	Palmeira et al., 2018
*mcr-*1.1	clinical	***E. coli* ST-46;**	China	Feng et al., 2018
*mcr-*1.1	clinical	***K. pneumoniae* ST-1296; *E. coli* ST-782**	Japan	Tada et al., 2018
*mcr-*1.1	public transport	*E. coli* ST-2253, *E. coli* ST-101, *E. coli* ST-10, *E. coli* ST-37	China	Shen et al., 2018
*mcr-*1.1	*Chrysoma* spp. flies	*K. pneumoniae* ST-43; *E. coli* ST-162; *E. coli* ST-1244; *E. coli* ST-10; *E. coli* ST-181; *E. coli* ST-549; *E. coli* ST-201; *E. coli* ST-218;	Thailand	Yang et al., 2019
*mcr-*1.1	ABF	*E. coli* ST-278	China	Bai et al., 2018
*mcr-*1.1	clinical	*E. coli* ST-744; *K. pneumoniae* ST-101	Brazil	Perdigão Neto et al., 2019
*mcr-*1.1	clinical	***E. coli* ST-10; *E. coli* ST-9; *E. coli* ST-5442**	Uruguay	Papa-Ezdra et al., 2020
*mcr-*1.1	shrimp	*V. parahaemolyticus* VP181	China	Lei et al., 2019
*mcr-*1.1	municipal wastewater	*E. coli* ST-131; *E. coli* ST-135; *E. coli* ST-764; *E. coli* ST-453; *E. coli* ST-10; *E. coli* ST-871; *E. coli* ST-457	Japan	Hayashi et al., 2019
*mcr-*1.1	poultry, pork and turkey meat	*S.* Typhimurium ST-19; *S.* Typhimurium ST-4556;	Brazil	Rau et al., 2020
*mcr-*1.1	raw retail chicken	*E. coli* ST-1169*; E. coli* ST-371; *E. coli* ST-156;	Egypt	Sadek et al., 2021
*mcr-*1.1	raw turkey products	*E. coli* ST-10*; E. coli* ST-744*; E. coli* ST-1079*; E. coli* ST-354*; E. coli* ST-349*; K. pneumoniae* ST-11; *K. pneumoniae* ST-147;	Czech Republic	Zelendova et al., 2020
*mcr-*1.1	ABF (poultry)	*E. coli* ST-155*; E. coli* ST-7458*; E. coli* ST-1140*;*	Lebanon	Kassem et al., 2021
*mcr-*1.1	healthy adults	***K. pneumoniae* ST-391; *K. pneumoniae* ST-37**;	China	Lu et al., 2020
*mcr-*1.1	ABF (pigs)	*E. coli* ST-746*; E. coli* ST-617*;*	China	Peng et al., 2019
*mcr-*1.1	fresh vegetables	*E. coli* ST-156;	China	Liu and Song, 2019
*mcr-*1.1	clinical (outpatients)	***E. coli* ST-206; *E. coli* ST-354**;	Brazil	Zamparette et al., 2020
*mcr-*1.1	pigs; white storks	*E. coli* ST-156*; E. coli* ST-10; *E. coli* ST-118; *E. coli* ST-224; *E. coli* ST-524; *E. coli* ST-42; *E. coli* ST-93; *E. coli* ST-1011;	Spain	Migura-Garcia et al., 2019
*mcr-*1.26 *mcr-*1.27	clinical	***E. coli* ST-155; *E. coli* ST-69;**	Germany	Neumann et al., 2020
*mcr-*1.1	retail meats	*E. coli* ST-38; *E. coli* ST-58; *E. coli* ST-443; *E. coli* ST-1737; *E. coli* ST-3889; *E. coli* ST-3998	South Korea	Kim et al., 2020
*mcr-*1.1	rainbow trout aquaculture	*E. coli* ST-48; *E. coli* ST-101;	Lebanon	Hassan et al., 2020
*mcr-*1.1	dog feces	*E. coli* ST-132;	China	Du et al., 2020
*mcr-*1.1	retail meats	*E. coli* ST-367; *E. coli* ST-716; *E. coli* ST-471; *E. coli* ST-310; *E. coli* ST-342; *E. coli* ST-86;	Belgium	Timmermans et al., 2021
*mcr-*2.1		*E. coli* ST-638;		
*mcr-*1.1	retail meats	*E. coli* ST-1630; *E. coli* ST-48; *E. coli* ST-617;	Laos	Moser et al., 2021
	traveler	*E. coli* ST-34; *E. coli* ST-10;		

*ABF – animal breeding farms; underlined and **bolded** strains originated from clinical sources.*

The first European environmental *mcr*-producing *E. coli* strain was obtained from Italian diarrhoeic veal calves in 2005 (Haenni et al., 2016), whereas the first European strain of clinical origin harboring plasmid-mediated colistin-resistance gene, was described in Denmark in a *Salmonella* Typhimurium ST34 strain with an *mcr-*3 variant (Litrup et al., 2017).

Here, we describe mobile pEtN transferase, *mcr*-1.1, encoded within a T4SS-containing 33.3 kbp IncX4 plasmid, pMUB-MCR. Similar IncX4 plasmids harboring different *mcr* variants were recently reported all over the world, with particular reference to animal breeding farms and environmental settings. Global dissemination of similar *mcr*-harboring IncX4 plasmids in Enterobacterales is presented in Table 6. So far, IncX4 *mcr*-harboring strains have been reported mainly in animal breeding farms, meat industry, and natural environments. Incidence of IncX4 *mcr*-harboring strains originating from clinical sources has been recently reported, in Switzerland (*E. coli* ST-5, ST-48), Portugal (*K. pneumoniae* ST-45, ST-1112, *Salmonella* spp.), Italy (*E. coli* ST-354; *K. pneumoniae* ST-512), France (*E. coli* ST-1288), Germany (*E. coli* ST-155; *E. coli* ST-69), Finland (*E. coli* ST-93), China (*i. a. E. coli* ST-2448; *E. coli* ST-167; *E. coli* ST-10), Brazil (*E. coli* ST-101; *K. pneumoniae* ST-437), United States of America (*E. coli* O157:H48), Thailand (*K. pneumoniae* ST-16; *K. pneumoniae* ST-45), Japan (*K. pneumoniae* ST-1296; *E. coli* ST-782), and United Kingdom (*S*. Typhimurium ST-34). Moreover, according to the current state of knowledge, *E. coli* is the major IncX4 clinical producer present in natural environments, animal breeding farms, as well as, in hospital settings. Recent research study performed by Zaja̧c et al. (2019) highlighted the importance of poultry farming, with particular emphasis on turkey, providing important reservoirs of *mcr*-1.1-carrying *E. coli* strains in Poland. The authors showed a wide diversity of IncX4 harboring strains, including 32 distinct sequence types (Table 6; Zaja̧c et al., 2019). Furthermore, the first clinical occurrence of a mcr-producing pathogen in Poland was reported by Izdebski et al. (2016). Clinical *E. coli* ST-617 strain, a member of ST-10 complex, possesses ∼250 kbp plasmid carrying *mcr*-1.1 and *bla*_*CMY–2*_-containing IncA/C2 plasmids (∼160 kbp).

In this study, we described the following strains – two ST-10, ST-93, ST-162, ST-553, and ST-6856. Interestingly, ST-553 and ST-162 strains have been recently described as *mcr-*producers in turkeys from animal breeding farms in Poland and Germany, while ST-93 have been already reported in clinical settings in Finland. Moreover, colistin-resistant IncX4-producing *E. coli* ST-10 seems to be widely distributed globally, and were already reported in Belgium (swine), Italy (river), Germany (barn dog feces), Poland (turkeys), Spain (swine), Czech Republic (raw turkey products), Brazil (wild birds; natural environment), Thailand (*Chrysoma* spp. flies), China (hospital setting; public transport), Uruguay (clinical source), and Japan (retail meat; municipal wastewater). This is in accordance with a recent report published by Matamoros et al. (2017), suggesting that *E. coli* ST-10 lineage, a sequence type known for its ubiquity in human fecal samples and in food samples, may function as an important reservoir of the *mcr-*1.1 gene (Matamoros et al., 2017).

The enormous genome plasticity of Gram-negative bacteria enables the accumulation of many different mechanisms of resistance to various antimicrobial agents. As a result, the increased emergence of MDR or XDR pathogens considerably reduces the opportunities for effective treatments against these bacteria (Livermore et al., 2011; Ojdana et al., 2015). A number of recent reports highlights the importance of *mcr* dissemination in clinical MDR bacteria, especially among subpopulations of pathogens persisting in hospital environments. Co-occurrence of *mcr* and ESBLs (CTX-M-15), different carbapenemases (KPC-type, OXA-181), and other antimicrobial resistance determinants may possibly lead to formation of pandrug-resistant bacterial lineages (Brauer et al., 2016; Di Pilato et al., 2016; Haenni et al., 2016; Caltagirone et al., 2017; Pulss et al., 2017; Tacão et al., 2017; Dalmolin et al., 2018; Mendes et al., 2018; Manageiro et al., 2019). In this study *mcr* coexisted with determinants of resistance to aminoglycosides [*aph*(3″)-Ib; *aph*(3″)-Ia; *aph*(6)-Id; *aadA1; aac(3″)-IIa; aadA2b*], chloramphenicol (*catA1, cmlA1)*, β-lactams (*bla*_*TEM–1A*_; *bla*_*TEM–1B*_; *bla*_*TEM–1D*_), quinolones (*qnrB19, qnrS1*), sulfonamides (*sul*1, *sul*2, *sul*3), and trimethoprim (*dfrA*1, *dfrA*14, *dfrA*15). Interestingly, in case of *E. coli* MIN11, *bla*_*TEM–1B*_ gene was present within the bacterial chromosome structure. Di Conza et al. (2014) proved that amoxicillin-clavulanate resistance with retained second-and third-generation cephalosporins susceptibility may be linked with *bla*_*TEM–1*_ overproduction (Di Conza et al., 2014). Furthermore, Salverda et al. (2010) have recently proved that several amino acid substitutions were also identified as factors involved in increased resistance to β-lactam-clavulanate. Interestingly, in the case of tested extraintestinal *E. coli* subpopulation, the only ciprofloxacin-susceptible strain MIN14, possessed a plasmid-borne *qnrS1* gene, which could be associated with low ciprofloxacin MICs. Allou et al. (2009) showed that *qnrS1*-possesing *E. coli* transconjugants showed low-level resistance to fluoroquinolones, with ciprofloxacin MIC ranging from 0.25 to 0.5 mg/L (Allou et al., 2009). In our study, *qnrS1*-producing *E. coli* MIN14 strain was classified as ciprofloxacin susceptible, with MIC ≤ 0.25 mg/L.

In conclusion, the increasing prevalence of plasmids responsible for colistin-resistance, often carrying other determinants of drug resistance, may possibly lead to formation of pandrug-resistant bacterial lineages. Great effort needs to be taken to avoid further dissemination of plasmid-mediated colistin resistance among clinically relevant Gram-negative pathogens.

## Data Availability Statement

The data presented in the study are deposited in the GenBank repository under BioProject number PRJNA700422 and accession numbers SAMN17831481 (*E. coli* MIN6—CP069692.1 for chromosome and CP069693.1–CP069700.1 for plasmids); SAMN17831482 (*E. coli* MIN9—CP069682.1 for chromosome and CP069683.1–CP069691.1 for plasmids); SAMN17831483 (*E. coli* MIN10—CP069677.1 for chromosome and CP069678.1–CP069681.1 for plasmids); SAMN17831484 (*E. coli* MIN11—CP069666.1 for chromosome and CP069667.1–CP069676.1 for plasmids); SAMN17831485 (*E. coli* MIN12—CP069657.1 for chromosome and CP069658.1–CP069665.1 for plasmids); and SAMN17831486 (*E. coli* MIN14—CP069646.1 for chromosome and CP069647.1–CP069656.1 for plasmids).

## Ethics Statement

This molecular investigation uses strains obtained from collection of strains deposited in Department of Microbiological Diagnostics and Infectious Immunology, Medical University of Bialystok, Poland. The Bioethics Commission of the Medical University in Bialystok did not require the study to be reviewed or approved by an ethics committee because apart from the strains from the Department’s collection, no data enabling patient identification was used in the study.

## Author Contributions

PiM, DS, JN, and ET substantially contributed to the conception of the submitted research manuscript, designing and validation of the experiments, and data acquisition and interpretation (antimicrobial susceptibility testing, short-read sequencing, long-read sequencing, and preparation of the figures and tables). PaM, AG, AS, and DS wrote the main manuscript. PiM was responsible for library preparation, WGS, and bioinformatics. PaM, DG, DI, PS, AZ, PR, PW, THa, IS, KM, RC, and JD contributed to the validation of the designed experiments and data acquisition and interpretation. THr, BK, JK, JG, SC, and PR were responsible for the medical care of the patient. All authors reviewed the manuscript.

## Conflict of Interest

The authors declare that the research was conducted in the absence of any commercial or financial relationships that could be construed as a potential conflict of interest.

## Publisher’s Note

All claims expressed in this article are solely those of the authors and do not necessarily represent those of their affiliated organizations, or those of the publisher, the editors and the reviewers. Any product that may be evaluated in this article, or claim that may be made by its manufacturer, is not guaranteed or endorsed by the publisher.

## Footnotes

1
https://itol.embl.de/
